# Modulation of Brain Activity and Functional Connectivity by Acupuncture Combined With Donepezil on Mild-to-Moderate Alzheimer's Disease: A Neuroimaging Pilot Study

**DOI:** 10.3389/fneur.2022.912923

**Published:** 2022-07-11

**Authors:** Yijun Zhan, Qinhui Fu, Jian Pei, Mingxia Fan, Qiurong Yu, Miao Guo, Houguang Zhou, Tao Wang, Liaoyao Wang, Yaoxin Chen

**Affiliations:** ^1^Department of Acupuncture, Longhua Hospital, Shanghai University of Traditional Chinese Medicine, Shanghai, China; ^2^Shanghai Key Laboratory of Magnetic Resonance, Department of Physics, East China Normal University, Shanghai, China; ^3^Department of Geriatrics, Huashan Hospital, Fudan University, Shanghai, China; ^4^Alzheimer's Disease and Related Disorders Center, Shanghai Mental Health Center, School of Medicine, Shanghai Jiaotong University, Shanghai, China

**Keywords:** Alzheimer's disease, acupuncture, Donepezil, functional magnetic resonance imaging (fMRI), fractional amplitude of low-frequency fluctuation (fALFF), functional connectivity (FC)

## Abstract

**Background:**

Functional brain imaging changes have been proven as potential pathophysiological targets in early-stage AD. Current longitudinal neuroimaging studies of AD treated by acupuncture, which is one of the growingly acknowledged non-pharmacological interventions, have neither adopted comprehensive acupuncture protocols, nor explored the changes after a complete treatment duration. Thus, the mechanisms of acupuncture effects remain not fully investigated.

**Objective:**

This study aimed to investigate the changes in spontaneous brain activity and functional connectivity and provide evidence for central mechanism of a 12-week acupuncture program on mild-to-moderate AD.

**Methods:**

A total of forty-four patients with mild-to-moderate AD and twenty-two age- and education-level-matched healthy subjects were enrolled in this study. The forty-four patients with AD received a 12-week intervention of either acupuncture combined with Donepezil (the treatment group) or Donepezil alone (the control group). The two groups received two functional magnetic resonance imaging (fMRI) scans before and after treatment. The healthy subject group underwent no intervention, and only one fMRI scan was performed after enrollment. The fractional amplitude of low-frequency fluctuation (fALFF) and functional connectivity (FC) were applied to analyze the imaging data. The correlations between the imaging indicators and the changed score of Alzheimer's Disease Assessment Scale-Cognitive Section (ADAS-cog) were also explored.

**Results:**

After the 12-week intervention, compared to those in the control group, patients with AD in the treatment group scored significantly lower on ADAS-cog value. Moreover, compared to healthy subjects, the areas where the fALFF value decreased in patients with AD were mainly located in the right inferior temporal gyrus, middle/inferior frontal gyrus, middle occipital gyrus, left precuneus, and bilateral superior temporal gyrus. Compared with the control group, the right precuneus demonstrated the greatest changed value of fALFF after the intervention in the treatment group. The difference in ADAS-cog after interventions was positively correlated with the difference in fALFF value in the left temporal lobe. Right precuneus-based FC analysis showed that the altered FC by the treatment group compared to the control group was mainly located in the bilateral middle temporal gyrus.

**Conclusion:**

The study revealed the key role of precuneus in the effect of the combination of acupuncture and Donepezil on mild-to-moderate AD for cognitive function, as well as its connection with middle temporal gyrus, which provided a potential treating target for AD.

**Trial Registration Number:**

NCT03810794 (http://www.clinicaltrials.gov).

## Introduction

Alzheimer's disease (AD), with high disability and mortality, leads to huge burden throughout the world (1, 2). To effectively break the progression of the disease, it is urgent to discover the key treating target for AD. Since the research diagnosis criteria of AD published by International Working Group (IWG)-2 in 2014 raised magnetic resonance imaging (MRI) as one of the topographical markers (3), studies based on functional magnetic resonance imaging (fMRI) technique have been laid growing emphasis on. Recently, functional brain imaging changes have been proven as potential targets in early-stage AD (4), among which fractional amplitude of low-frequency fluctuation (fALFF) and functional connectivity (FC) are the most commonly used imaging indexes. Both fALFF, directly reflecting spontaneous brain activity, and FC decreased as early as in the preclinical AD stage of subjective cognitive decline (5). A study has carried out combined analysis of fALFF with FC, seeking precisely-significant seed point through the former method and applying it in the latter analysis, to investigate the pathological mechanisms of AD as rigorously as possible (5). Several brain regions, including the hippocampus, posterior cingulate cortex, precuneus, pre-frontal cortex, temporal lobe, and angular gyrus, have been reported and well known as the core brain regions involved in the pathophysiology of AD (6–8).

In view of the huge challenges the new drug development for AD is now facing, the effects of non-pharmacological interventions are gaining attention. Our previous review has showed that acupuncture therapy, exercise intervention, and repetitive transcranial magnetic stimulation could bring benefits to patients with AD not only in cognition function, but also in daily living ability and life quality (9). Specifically, acupuncture, with a history of over 3,000 years, is gradually acknowledged in its therapeutic effects and regulation of neuroplasticity in AD (10).

When it comes to the mechanisms of acupuncture treating AD, neuroimaging studies in the recent decade have demonstrated that acupuncture could induce positive modulation of specific brain regions and neuroplastic reorganization of brain functional networks in AD or mild cognitive impairment (11–14). There were several regions that show activated changes in patients with MCI and AD after immediate manual or electro-acupuncture stimulation, including the default-mode network (DMN), left frontal parietal network, right frontal parietal network, visual network, sensorimotor network, and auditory network (15). Moreover, there is certain correlation between the changes in cognitive function and alteration in functional connectivity. However, low consistency revealed in current research results, due to the differences in study designs, scanning parameters, and selection of seed point. Current neuroimaging studies of acupuncture for AD have not adopted comprehensive acupuncture protocols, nor have explored the changes after a complete treatment duration. As a part of a multicenter randomized controlled trial, this neuroimaging pilot study was carried out to obtain scientific and objective evidence for the central mechanism of acupuncture program combined with Donepezil on AD.

## Materials and Methods

### Participants

This pilot study was approved by the Medical Ethics Committee of Longhua Hospital, Shanghai University of Traditional Chinese Medicine (no. 2018LCSY060). In this study, 44 right-handed patients with AD and 22 age-, gender-, and education-matched healthy subjects were enrolled. The patients were recruited from March 2019 to December 2020 at the Department of Acupuncture Longhua Hospital, Shanghai University of Traditional Chinese Medicine, the Department of Geriatrics, Huashan Hospital, Fudan University, and Alzheimer's Disease and Related Disorders Center, the Department of Geriatrics, Shanghai Mental Health Center, School of Medicine, Shanghai Jiaotong University. The healthy subjects were recruited from the local community by advertisements.

Patients with AD met the diagnostic criteria of Neurological Communicative Disorders and Stroke and the Alzheimer's Disease and Related Disorders Association (NINCDS-ADRDA) for AD, with Mini-Mental State Examination (MMSE) evaluated as mild-to-moderate AD (11 to 22 for primary school education, 11 to 26 for middle school education, or above). The patients' scale for medial temporal lobe atrophy (MTA-scale) was required to be no <2 for those under 75 years old, and no <3 for those over 75 years old. Exclusion criteria for patients with AD included cognitive impairment caused by other factors, such as frontotemporal dementia, dementia with Lewy bodies, vascular dementia, severe depression, cerebrovascular disease, tumors, poisoning, metabolic diseases, and infections; severe chronic disorders, such as Parkinson's disease, epilepsy, myocardial infarction, heart failure, malignant tumor, severe infection, impaired hepatic or renal function; aphasia, disturbance of consciousness, or failure to cooperate with the related examinations due to physical disability; anticoagulant treatments, such as warfarin or heparin; and the use of pacemakers or receiving acupuncture in the past 2 weeks.

The matched healthy subjects had a normal cognitive function, with MMSE evaluated as above 27, whereas subjects with severe chronic disorders mentioned above were excluded.

For all the participants, those with contraindications to undergoing an MRI scan should be excluded, such as claustrophobia or pacemaker implantation. Written informed consent was provided by every participant.

### Interventions

The 44 patients with AD were randomly assigned into either the treatment group (acupuncture combined with Donepezil) or the control group (Donepezil along). Both groups took Donepezil 5 mg one time daily before bed time for 12 weeks.

Patients in the treatment group received additional acupuncture treatment. Disposable stainless steel needles (0.25 mm × 25 mm) were used. Based on our previous studies on the acupoint selection and the clinical experience (9), the main acupoints of this study were Shenting (DU24), Yintang (EX-HN3), Baihui (DU20), Sishencong (EX-HN1), Wangu (GB12), Shenmen (HT7), Zhaohai (KI6), and Xuanzhong (GB39). The detailed information for the locations of the acupoints is presented in Supplementary Table S1. Among them, DU24 and GB12 were electrically stimulated as a pair, with a disperse-dense wave of 2/50 Hz, 0.5 mA. The treatment included thirty-six 30-min sessions over 12 weeks.

### Resting-State fMRI Imaging Acquisition

Image acquisitions were performed in Shanghai Key Laboratory of Magnetic Resonance on a 3T MR scanner (Discovery MR750 3.0T scanner, GE Healthcare, US) with a 64 channel head coil. A high-resolution three-dimensional T1-weighted imaging was performed with the following parameters: slice number = 192, slice thickness = 1 mm, repetition time (TR) = 2,530 ms, echo time (TE) = 2.98 ms, field of view (FOV) = 224 mm × 224 mm, gap = 0 mm, acquisition matrix = 64 × 64.

Resting-state data were performed with the following parameters: slice number = 35, slice thickness = 3.5 mm, repetition time (TR) = 500 ms, echo time (TE) = 30 ms, field of view (FOV) = 224 mm × 224 mm, gap = 0.525 mm, acquisition matrix = 64 × 64, flip angle = 60°, total time = 8 min 7 s.

During the scan, participants were asked to relax with their eyes closed but not to fall asleep. Images of patients with AD were acquired at baseline and week 12, respectively. Healthy subjects received the fMRI scan only one time at baseline.

### Data Preprocessing

Preprocessing of the rs-fMRI data was performed using SPM12 (http://www.fil.ion.ucl.ac.uk/spm/) and Data Processing Assistant for Resting-State fMRI (DPARSF) (16). The first 40 volumes were discarded from a total of 960 volumes to allow for signal equilibrium of the initial magnetic resonance signals and adaptation of the subjects to the circumstances. Then, slice timing correction was carried out; realignment for head-motion correction, coregistration of the functional images to DARTEL template, spatial normalization to the Montreal Neurological Institute (MNI) 24 template (resampling voxel size = 3 mm × 3 mm × 3 mm), and smoothing with an isotropic Gaussian kernel (full width at half maximum = 8 mm), detrending and regression out covariables were performed in order. Any subject with a head motion >2.0-mm translation or a 2.0° rotation in any direction was excluded.

### Primary Measurements

#### fALFF Analysis

In this study, the DPARSF software package was used to calculate the fALFF value on MATLAB R2016a. The calculation principle was used to transform the time series of the image into the frequency domain through fast Fourier transform and obtain the power spectrum. At each frequency point, the square root of the power spectrum was calculated, and then, the average square root was obtained at 0.01–0.08 Hz of each voxel. The fALFF value of the image was calculated as the ratio of the low-frequency power spectrum to the power spectrum of the whole frequency range.

#### Seed-Based FC Analysis

The FC analysis method based on seed points was used to explore the features of functional connectivity of the brain regions of interest in patients with AD. Before conducting FC, the images were filtered between 0.01 and 0.08 Hz to control noise interference. The brain area most significantly related to the AD in the fALFF analysis result was selected as the seed point, and the DPARSF software package was applied to perform the whole-brain FC analysis on the MATLAB R2016a platform.

### Secondary Measurement

#### Alzheimer's Disease Assessment Scale-Cognitive Section (ADAS-Cog)

Alzheimer's Disease Assessment Scale-Cognitive Section is a general neuropsychological measurement tool for evaluating cognitive function (17), which is the most widely used scale in clinical trials for AD. It can keenly identify the cognitive changes and therapeutic effects of subjects with mild-to-moderate AD. The main evaluation indicators include 11 cognitive and 1 non-cognitive items. The highest score is 70 points. The higher the score, the worse the cognitive function.

### Statistical Analysis

The statistical analysis of demographic characteristics and clinical measurements was carried out using the Statistical Packing for the Social Sciences (SPSS) version 19.0 (IBM Corp., Armonk, NY, USA). The analysis of variance (ANOVA) was used for continuous variable, and the chi-square test was used for categorical variables.

SPM12 software was used to carry out the statistical analysis of fALFF and FC. The one-way ANOVA was used to analyze the differences between different groups. The statistical significance level was set as *p* < 0.001. The false discovery rate (FDR) or family-wise error (FWE) theory was used for multiple comparison corrections (voxel-wise *p* < 0.01, cluster-wise *p* < 0.05). *Post hoc* analysis was also performed with the Bonferroni correction to evaluate the differences between the different groups (*p* < 0.05). Taking gender, age, and education level as covariates, multiple regression analysis was used to analyze the correlation between the changes of fALFF and FC with the differences in ADAS-cog before and after treatment in patients with AD. The xjview software (http://www.alivelearn.net/xjview) was utilized to display the results as images.

## Results

### Demographic and Clinical Information

A total of 44 patients with AD and 22 healthy subjects were included in the study. The subjects in the treatment group and control group completed all the treatment procedures. Then, 1 case in healthy subject group was terminated due to the tendency of claustrophobia during the scan. In preprocessing period, 2 cases in the treatment group, 2 cases in the control group, and 1 case in the healthy subject group were excluded because of head movement translation> 2 mm and/or rotation>2°. Finally, 20 cases in each group were included in the statistical analysis. Demographic and clinical characteristics of these participants are shown in Table 1. There were no significant differences in age, education level, personal history, and past history across the three groups (Table 1).

**Table 1:** Baseline characteristics of participants.

**Characteristics**	**Treatment group**	**Control group**	**Healthy subject group**
	**(*N* = 20)**	**(*N* = 20)**	**(*N* = 20)**
**Age, years (mean ± SD)**	61.82 ± 6.118	60.52 ± 7.420	60.97 ± 7.274
**Sex, male [count (%)]**	9 (45.0%)	8 (40.0%)	10 (50.0%)
**Course, months [M(IQR)]**	15 (12)	14 (14)	/
**Education level [count (%)]**			
illiteracy	2 (10.0%)	2 (10.0%)	2 (10.0%)
Primary school	5 (25.0%)	3 (15.0%)	4 (20.0%)
Middle school	4 (20.0%)	7 (35.0%)	5 (25.0%)
High school	5 (25.0%)	3 (15.0%)	4 (20.0%)
College	4 (20.0%)	5 (25.0%)	5 (25.0%)
**Past medical history [count(%)]**			
Hypertension	9 (45.0%)	10 (50.0%)	9 (45.0%)
Heart disease	4 (20.0%)	5 (25.0%)	4 (20.0%)
Diabetes	5 (25.0%)	5 (25.0%)	4 (20.0%)
Hyperlipidemia	8 (40.0%)	7 (35.0%)	7 (35.0%)
**Alcohol [count (%)]**	3 (15.0%)	2 (10.0%)	3 (15.0%)
**Tobacco [count (%)]**	5 (25.0%)	4 (20.0%)	4 (20.0%)
**MMSE [M(IQR)]**	17 (5)	18 (6)	27 (4)

### Group Differences in fALFF

#### The Difference in fALFF Value Between Patients With AD and Healthy Subjects

**Figure 1:**
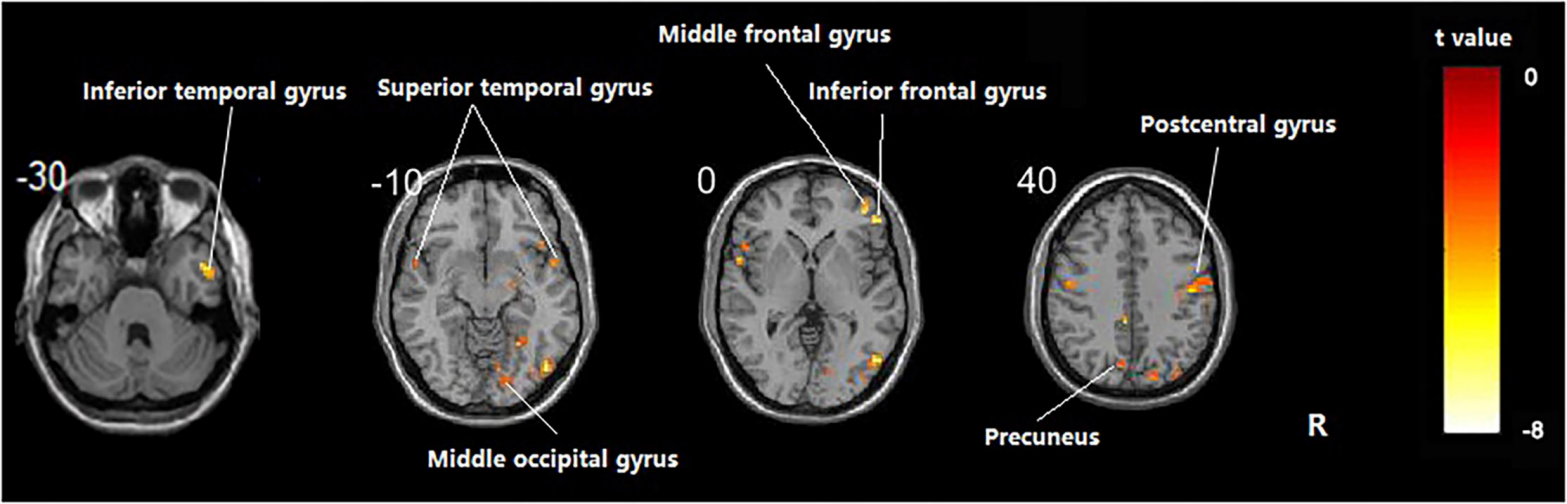
Regions showing significant fALFF value differences between healthy subjects and AD. The results were FDR-corrected with *p* < 0.05.

Compared with the healthy subject, decreased fALFF values of patients with AD were detected in the right inferior temporal gyrus, middle/inferior frontal gyrus, postcentral gyrus, middle occipital gyrus; the left precuneus; and the bilateral superior temporal gyrus (Table 2 and Figure 1, *p* < 0.05, FDR-corrected).

**Table 2:** Regions showing significant fALFF value differences between healthy subject and AD.

**Brain region**	**R/L**	**BA**	**MNI (Peak point)**			**T value**	**Voxel**
			**X**	**Y**	**Z**
Inferior temporal gyrus	R	37	48	−70	−4	−7.03	68
Inferior frontal gyrus	R	/	45	5	26	−6.63	16
Superior temporal gyrus	R	22	66	−43	11	−5.94	22
Postcentral gyrus	R	3	45	−22	53	−5.95	155
Middle frontal gyrus	R	6	30	−1	50	−5.54	30
Middle occipital gyrus	R	19	39	−79	8	−5.01	18
Superior temporal gyrus	L	/	−57	8	2	−4.83	14
Precuneus	L	/	−9	−73	35	−4.76	14

*R, right; L, left; MNI, Montreal Neurological Institute. p < 0.05, FDR–corrected*.

#### Comparison of fALFF Difference Within the Treatment Group and Control Group Before and After Intervention

It was shown that significant differences in fALFF value before and after intervention in the treatment group were located in the right precuneus, middle frontal gyrus, and superior temporal gyrus; and those in the control group were located in the right lingual gyrus and middle frontal gyrus and the left inferior temporal gyrus, lingual gyrus, and thalamus (Supplementary Tables S2, S3, Supplementary Figures S1, S2, *p* < 0.05, FDR-corrected).

#### Comparison of fALFF Difference Between the Treatment Group and Control Group Before and After Intervention

**Figure 2:**
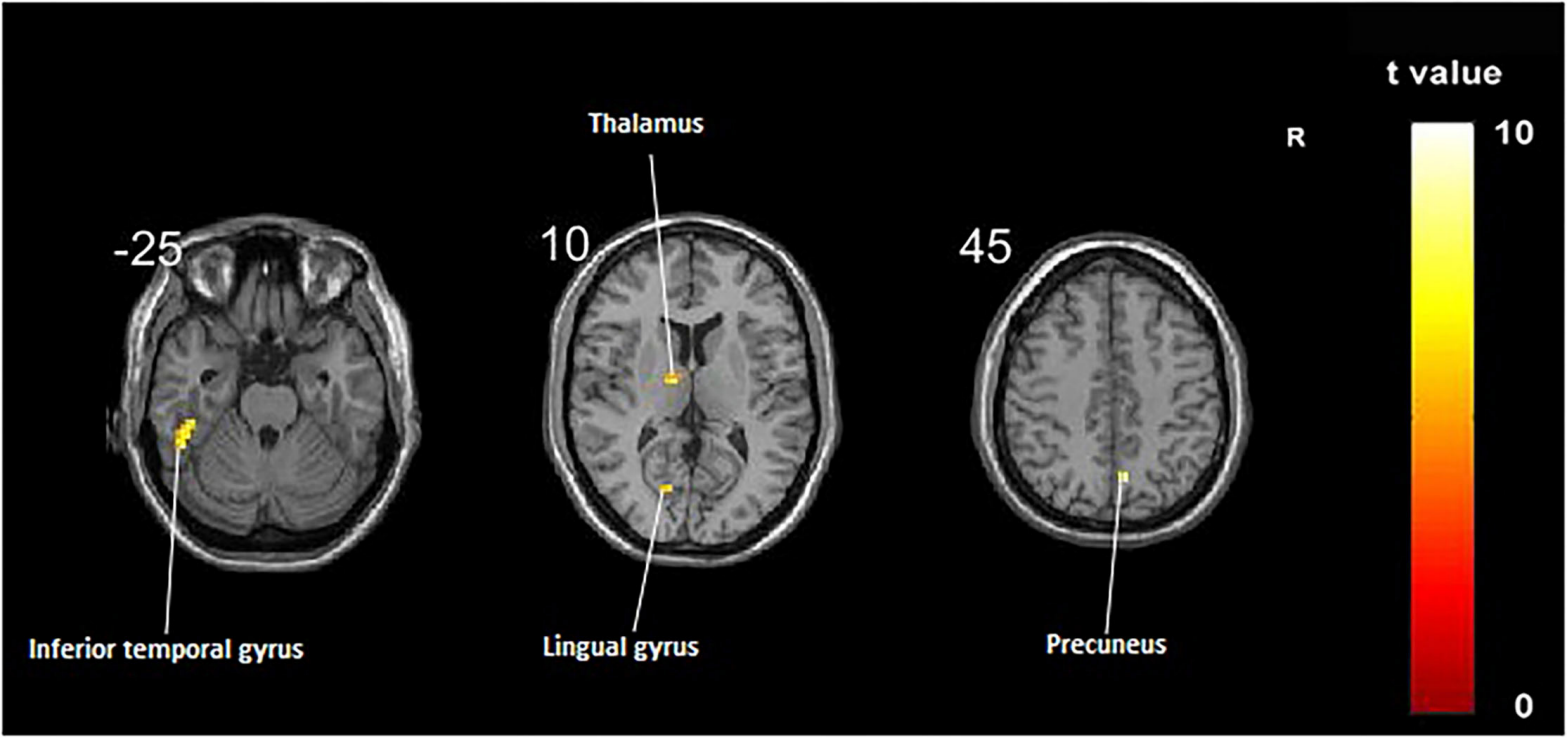
Regions showing significant fALFF value changes between the treatment group and control group. The results were uncorrected with *p* < 0.001.

Compared with the control group, significant differences in fALFF value before and after intervention in the treatment group were located in the right precuneus and the left inferior temporal gyrus, lingual gyrus, and thalamus, whereas did not pass the FDR or FWE correction (Table 3, Figure 2, *p* < 0.001, uncorrected).

**Table 3:** Regions showing significant fALFF value changes between the treatment group and control group.

**Brain region**	**R/L**	**BA**	**MNI (Peak point)**			***T*** **-value**	**Voxel**
			**X**	**Y**	**Z**
Precuneus	R	/	6	−58	65	8.94	15
Inferior temporal gyrus	L	/	−51	−46	−25	8.08	27
Lingual gyrus	L	/	−18	−82	−22	7.89	9
Thalamus	L	/	−12	−10	11	7.30	6

*R, right; L, left; MNI, Montreal Neurological Institute. p < 0.001, uncorrected*.

#### Correlation Analysis Between fALFF Difference and ADAS-Cog Difference in Treatment Group

The differences in ADAS-cog before and after the intervention within and between the treatment group and control group groups were both statistically significant (Table 4, *p* < 0.05).

**Table 4:** Change in ADAS–cog values within and between groups (mean ± SD).

	**Treatment group (*n* = 20)**	**Control group (*n* = 20)**
Week 0	19.32 ± 1.89	19.98 ± 1.59
Week 12	16.52 ± 1.47^a^^b^	18.08 ± 1.49^a^
ΔWeek 12–Week 0	2.80 ± 1.51^b^	1.90 ± 1.36

a *p < 0.05 within group;*

b *p < 0.05 between groups*.

**Figure 3:**
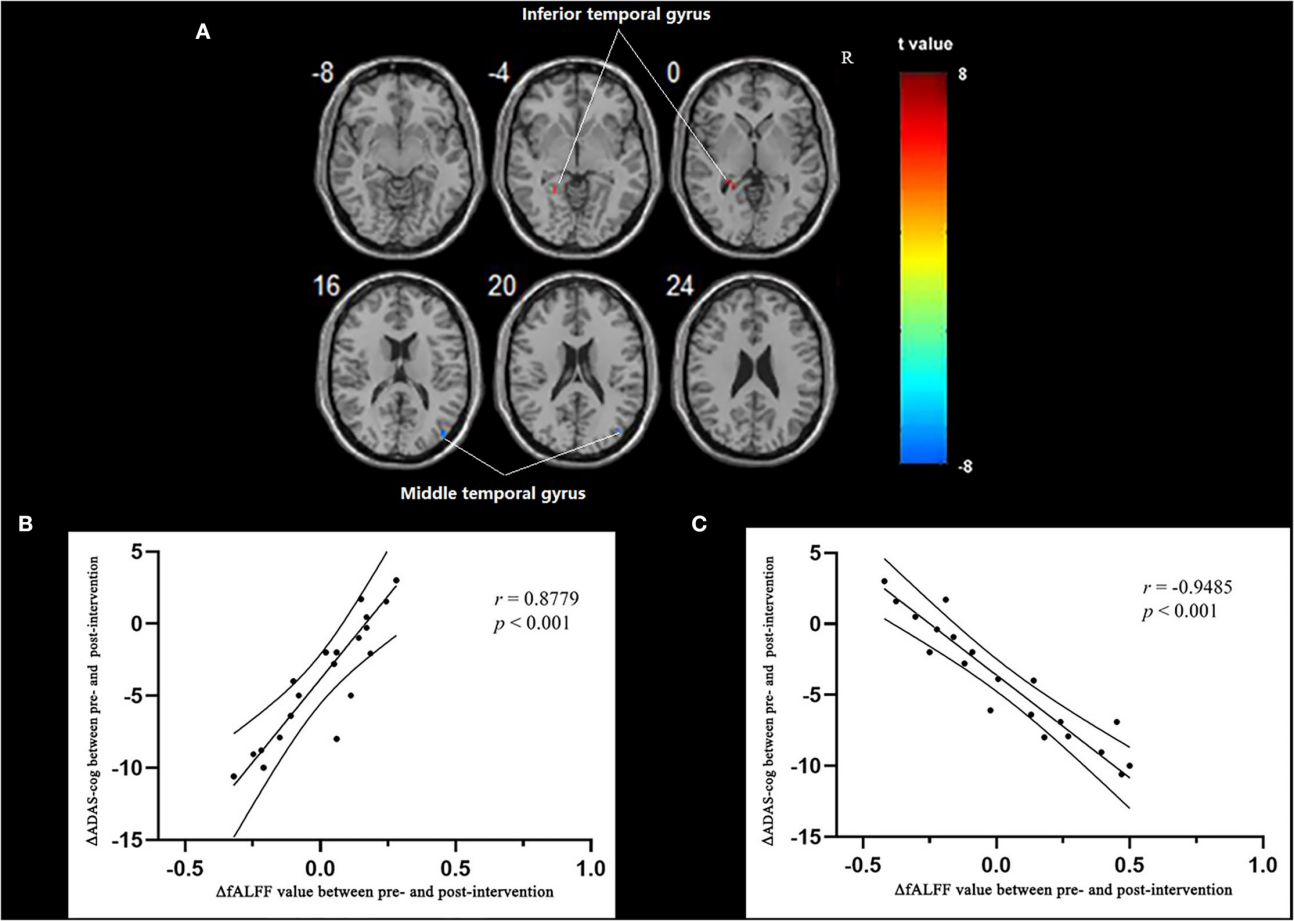
Regions showing significant correlation in the change in fALFF in the treatment group to the change in ADAS-cog. **(A)** Multiple regression correlation results of significant correlation between fALFF value changes and ADAS-cog value changes in the treatment group (*p* < 0.001, FWE-corrected). **(B)** Pearson correlation results of the change in fALFF value of the left inferior temporal gyrus and the change in ADAS-cog value. **(C)** Pearson correlation results of the change in fALFF value of the right middle temporal gyrus and the change in ADAS-cog value.

Multiple regression analysis, with covariates of age, gender, and educational level removed, showed that ADAS-cog difference caused by acupuncture combined with Donepezil was positively correlated with the change in ADAS-cog value in the left inferior temporal gyrus (*r* = 0.8779, *p* < 0.001, FWE-corrected) and was negatively correlated with the right middle temporal gyrus (*r* = −0.9485, *p* < 0.001, FWE-corrected) (Table 5, Figure 3).

**Table 5:** Regions showing significant correlation in the change in fALFF in the treatment group to the change in ADAS–cog.

**Brain region**	**R/L**	**BA**	**MNI (Peak point)**			***T*** **-value**	**Voxel**
			**X**	**Y**	**Z**
Inferior temporal gyrus	L	/	−24	−45	0	7.57	7
Middle temporal gyrus	R	/	42	−78	21	−5.06	6

*R, right; L, left; MNI, Montreal Neurological Institute. p < 0.05, FWE–corrected*.

### Group Differences in FC

#### Selection of Seed Points

**Figure 4:**
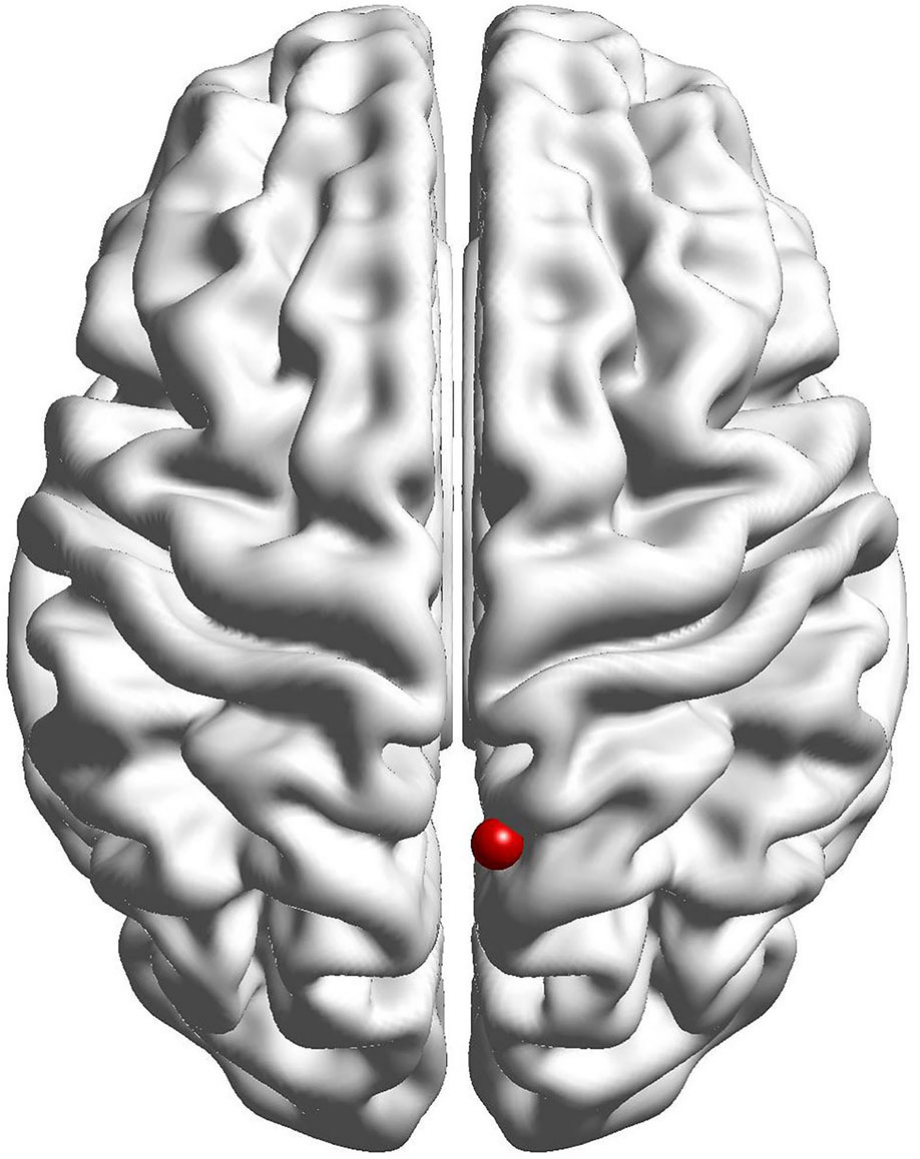
Seed point of the right precuneus for functional connection analysis.

The results of the fALFF analysis above revealed that the right precuneus was the significant point in the effect of acupuncture combined with Donepezil, as well as in the comparison between the treatment group and the control group. It was then hypothesized that the right precuneus might be the key point in the treatment of AD. Therefore, it was selected as the seed point to analyze the FC with the whole brain, locating at the MNI coordinates (6, −58, 65), with the radius of 6 mm, which was the frequently-used volume for a seed point (Figure 4).

#### Comparison of FC Difference Between the Treatment Group and Control Group Before and After Intervention

**Figure 5:**
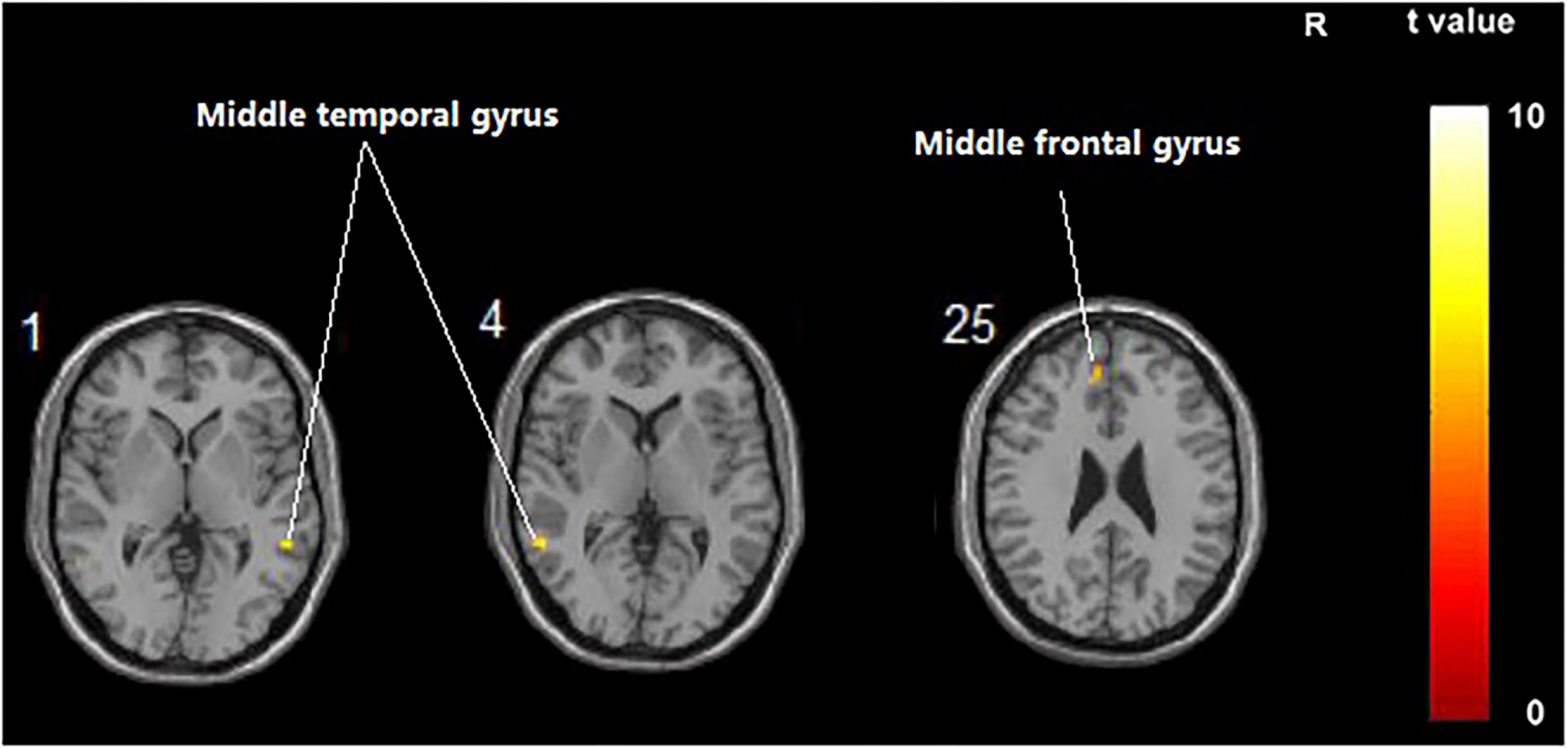
Regions showing significant FC changes between the treatment group and control group. The results were uncorrected with *5* < 0.001.

Compared with the control group, significant differences in FC value before and after intervention in the treatment group were located in the bilateral middle temporal gyrus and left middle frontal gyrus, whereas did not pass the FDR or FWE correction (Table 6, Figure 5, *p* < 0.001, uncorrected).

**Table 6:** Regions showing significant FC changes between the treatment group and control group.

**Brain region**	**R/L**	**BA**	**MNI (Peak point)**			***T*** **-value**	**Voxel**
			**X**	**Y**	**Z**
Middle temporal gyrus	R	/	51	−42	−3	10.84	5
Middle temporal gyrus	L	21	−57	−48	3	7.86	5
Middle frontal gyrus	L	/	−12	42	24	6.15	7

*R, right; L, left; MNI; Montreal Neurological Institute. p < 0.001, uncorrected*.

#### Correlation of Functional Connection of the Whole Brain and Right Precuneus and Cognitive Function Improvement in the Treatment Group

**Figure 6:**
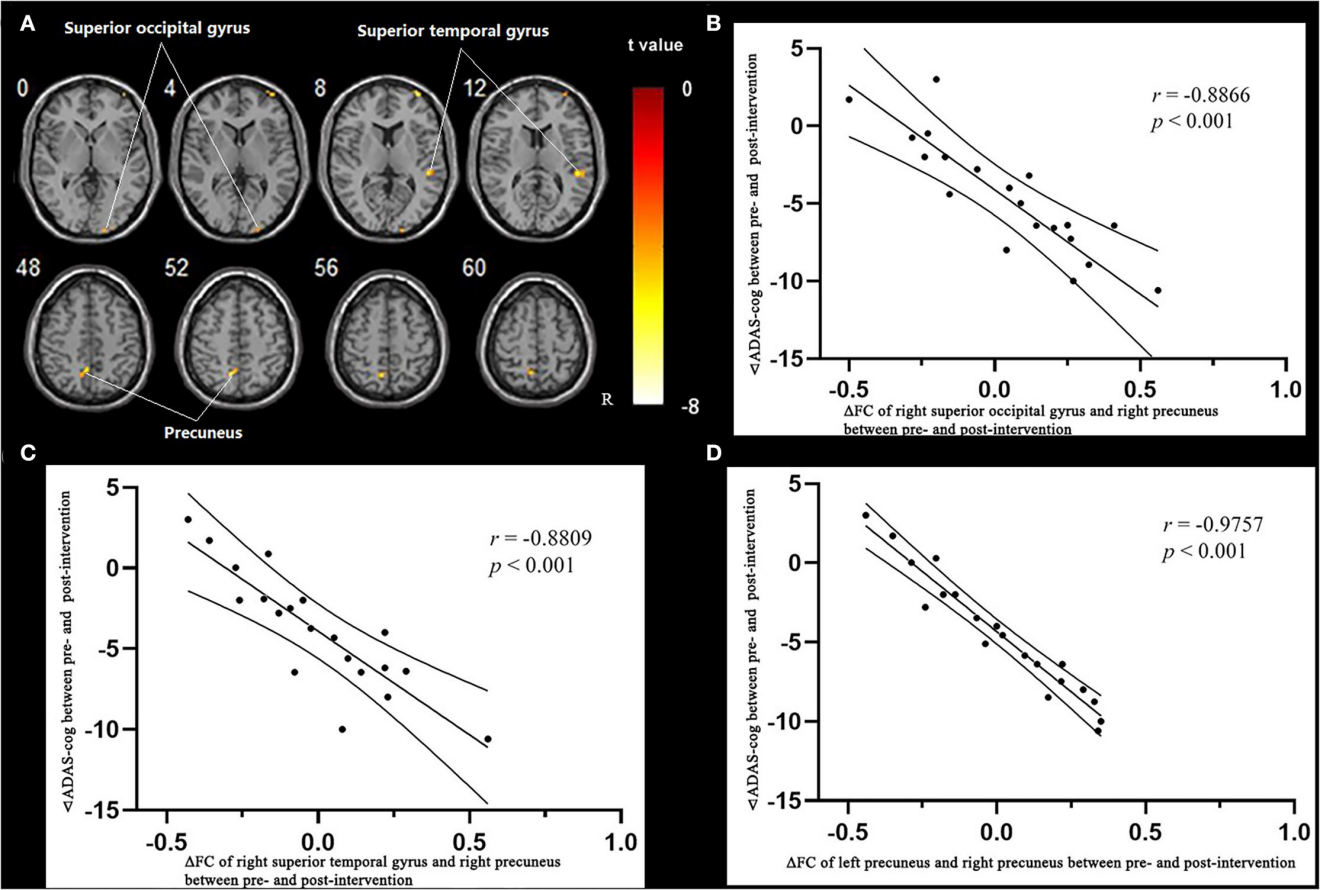
Regions showing significant correlation in the change in FC in the treatment group to the change in ADAS-cog. **(A)** Multiple regression correlation results of significant correlation between FC changes and ADAS-cog value changes in the treatment group (*p* < 0.001, FDR-corrected). **(B)** Pearson correlation results of the change in FC to the right superior occipital gyrus and the change in ADAS-cog value. **(C)** Pearson correlation results of the change in FC to the right superior temporal gyrus and the change in ADAS-cog value. **(D)** Pearson correlation results of the change in FC to the right superior temporal gyrus and the change in ADAS-cog value.

Multiple regression analysis, with covariates of age, gender, and educational level removed, showed that ADAS-cog difference caused by acupuncture combined with Donepezil was negatively correlated with the right precuneus-based FC of the right superior temporal gyrus (*r* = −0.8809, *p* < 0.001, FDR-corrected), right superior occipital gyrus (r = −0.8866, *p* < 0.001, FDR-corrected), and left precuneus (*r* = −0.9757, *p* < 0.001, FDR-corrected) (Table 7, Figure 6).

**Table 7:** Regions showing significant correlation in the change in FC in the treatment group to the change in ADAS–cog.

**Brain region**	**R/L**	**BA**	**MNI (Peak point)**			***T*** **-value**	**Voxel**
			**X**	**Y**	**Z**
Superior occipital gyrus	R	/	21	−102	6	−4.42	12
Superior temporal gyrus	R	41	48	−33	9	−6.07	18
Precuneus	L	/	−12	−57	51	−8.00	34

*R, right; L, left; MNI, Montreal Neurological Institute. p < 0.001, FDR–corrected*.

## Discussion

Our study implied that the treatment of acupuncture combined with Donepezil on patients with AD could mainly upregulate the abnormally decreased spontaneous neural activity of the left precuneus and its functional connectivity with the temporal gyrus, which were correlated with the improvement in cognitive function. Our results, to some extent, provide a potential brain region target for precise therapies, such as transcranial magnetic stimulation (TMS). Moreover, the brain region could be taken as the target for evaluating the effect of various therapies. The conclusions drawn in this study are basically consistent with the results of existing AD pathology studies. Decreased fALFF values were often detected in the precuneus, angular gyrus, hippocampus, superior frontal gyrus, paracentral lobule, occipitotemporal cortex, parietal lobule, etc. (18–20) In a cross-sectional study, patients with AD showed a clear reduction of cortical gray matter in the temporal lobe, precuneus, cingulate gyrus, and inferior frontal gyrus (21). These brain regions are at the center of many brain function networks and are closely related to the progression of AD (22). As the condition develops, the functional connectivity of the above brain areas is gradually weakened (23).

The precuneus and temporal gyrus are the main brain regions of the default-mode network, closely related to AD cognitive function. FC disruption within this network is associated with cognitive decline in patients. Then, the damage of DMN can appear in the early period of AD, which is manifested as the decrease in network connection and the decline of network integrity (24, 25).

The precuneus has been confirmed to demonstrate structural or functional abnormalities in patients with AD, such as cortical atrophy and metabolic impairment (26, 27). Pathologically, the precuneus is involved in Braak's IV and V stages, closely related to the process of AD from the appearance of the initial symptoms to the end period. The precuneus was also found to be involved in the tau deposition pathology of AD. Positron emission tomography (PET) studies were compared and analyzed to reveal that the tau pathology network (TPN) overlapped with DMN, and the tau distribution peak in the TPN was located in the precuneus (28). Recent research raised that the precuneus might be dually involved in both memory and perceptual metacognition due to the close relationship shared between the precuneus and perceptual metacognition (29, 30).

The temporal lobe, including the structure of hippocampus, is well accepted as one of the most critical brain regions relating to AD. Connectivity changes in the posterior-medial and anterior-temporal hippocampal networks were found to contribute together to the cognitive decline in Alzheimer's disease (31), which were proved to be key in episodic memory formation (32). Studies based on dynamic functional connectivity showed that the continuous cognitive impairment in AD leads to a gradual loss of meta-stable state in the whole brain, and the brain regions with significantly reduced effective functional connectivity were mainly located in the temporal lobe (33). Another acupuncture study on AD showed that the intervention could improve cognitive function by increasing the neuronal activity of the low frontotemporal lobe, especially the hippocampus (34).

Another finding of our study was that the increased FC between left and right precuneus correlated with the improvement in cognitive function by acupuncture combined with Donepezil, which could be explained by the functional region transfer by corpus callosum. A similar result had been drawn by an experiment of acupuncture on mouse models of vascular dementia, which implied the effect of acupuncture's ameliorating white matter damage in the corpus callosum (35). A previous study had shown that the significant alterations in white matter microstructural metrics caused by AD could be detected by the technology of diffusion tensor imaging (DTI), and the alteration indexes were closely related to the cognitive function degree of AD (36). The results above hint that further studies might apply DTI technology to investigate and prove the effect of acupuncture on the diffusivity of white matter fiber tracts.

## Limitations

A precise parameter for electroacupuncture has not been taken into consideration, which plays a key role in the clinical effect. A study has implied that high-frequency electrical acupuncture has a stronger protective effect on hippocampal synaptic plasticity and spatial learning and memory ability in AD rats, compared with low-frequency or intermediate-frequency stimulation (37). It is suggested that the crucial factors for the clinical effect and mechanism of acupuncture on AD should be included in the future study designs, such as frequency and intensity of electro-/manual acupuncture, and even the evaluation of *Deqi*.

## Conclusion

The central mechanism of acupuncture combined with Donepezil improving the cognitive dysfunction of patients with AD lied in the regulation of the abnormal reduction in spontaneous neuron activity and functional connectivity in the precuneus and temporal gyrus, which provided a potential treating target for AD, as well as a target with monitoring value in the progression of acupuncture intervention on AD.

## Data Availability Statement

The raw data supporting the conclusions of this article will be made available by the authors, without undue reservation.

## Ethics Statement

The studies involving human participants were reviewed and approved by the Medical Ethics Committee of Longhua Hospital, Shanghai University of Traditional Chinese Medicine. The patients/participants provided their written informed consent to participate in this study.

## Author Contributions

JP, YZ, and MF designed this study. QF, HZ, and TW collected and analyzed the clinical data. LW and YC scanned the imaging data. YZ, MF, QY, and MG analyzed the imaging data. YZ and QF wrote the manuscript. All authors contributed to the article and approved the submitted version.

## Funding

This work was supported by the key Scientific Research Program of the Science and Technology Commission of Shanghai Municipality (18401970500), the Financing Scheme of Arising Interdisciplinary Subjects of TCM of Shanghai Municipal Health Bureau (Shxxjcxk201709), the TCM genre program of Shanghai Municipal Health Bureau [YZ (2018–2020)-CCCX-1006], and the National Natural Science Foundation of China (81603697).

## Conflict of Interest

The authors declare that the research was conducted in the absence of any commercial or financial relationships that could be construed as a potential conflict of interest.

## Publisher's Note

All claims expressed in this article are solely those of the authors and do not necessarily represent those of their affiliated organizations, or those of the publisher, the editors and the reviewers. Any product that may be evaluated in this article, or claim that may be made by its manufacturer, is not guaranteed or endorsed by the publisher.

## Supplementary Material

Supplementary Figure S1 Regions showing significant fALFF value changes within the treatment group before and after treatment. The results were corrected with *p* < 0.05, FDR-corrected.

Supplementary Figure S2 Regions showing significant fALFF value changes within the control group before and after treatment. The results were corrected with *p* < 0.05, FDR-corrected.

## Abbreviations

AD: Alzheimer's disease
ADAS-cog: Alzheimer's Disease Assessment Scale-Cognitive Section
DMN: default-mode network
DPARSF: Data Processing Assistant for Resting-State fMRI
DTI: diffusion tensor imaging
fALFF: fractional amplitude of low-frequency fluctuation
FC: functional connectivity
FOV: field of view
MNI: Montreal Neurological Institute
MTA-scale: medial temporal lobe atrophy
NINCDS-ADRDA: Neurological Communicative Disorders and Stroke and the Alzheimer's Disease and Related Disorders Association
PET: positron emission tomography
SD: standard deviation
SPSS: Statistical Packing for the Social Sciences
TE: echo time
TPN: tau pathology network
TR: repetition time.
