# Supplementary material for: Modulation of Brain Activity and Functional Connectivity by Acupuncture Combined With Donepezil on Mild-to-Moderate Alzheimer's Disease: A Neuroimaging Pilot Study
Source: Front Neurol. 2022 Jul 11;13:912923. doi: 10.3389/fneur.2022.912923 (PMC9309357; doi:10.3389/fneur.2022.912923)
Supplement: Supplementary file 1 [file Table_1.DOCX]

SUPPLE Table 1 The locations of the acupoints selected

| Acupoint | Location |
| --- | --- |
| Shenting (DU24) | On the head, 0.5 cun directly above the midpoint of the anterior hairline. |
| Yintang (EX-HN3) | At the forehead, at the midpoint between the two medial ends of the eyebrow. |
| Baihui (DU20) | On the head, 5.0 cun directly above the midpoint of the anterior hairline. |
| Sishencong (EX-HN1) | At the vertex of the head, a group of four points, 1.0 cun respectively anterior, posterior and lateral to DU 20. |
| Wangu (GB12) | On the head, posterior to the auricle, in the depression posterior and inferior to the mastoid process. |
| Shenmen (HT7) | On the wrist, at the ulnar end of the transverse crease of the writs, in the depression on the radial side of the tendon m. flexor carpi ulnaris. |
| Xuanzhong (GB39) | On the lateral aspect of the lower leg, 3 cun above the tip of the external malleolus, on the anterior border of the fibula. |
| Zhaohai (KI6) | On the medial aspect of the foot, in the depression below the tip of the medial malleolus. |
