# Supplementary material for: Modulation of Brain Activity and Functional Connectivity by Acupuncture Combined With Donepezil on Mild-to-Moderate Alzheimer's Disease: A Neuroimaging Pilot Study
Source: Front Neurol. 2022 Jul 11;13:912923. doi: 10.3389/fneur.2022.912923 (PMC9309357; doi:10.3389/fneur.2022.912923)
Supplement: Supplementary file 2 [file Table_2.DOCX]

SUPPLE Table 2 Regions showing significant fALFF value changes within the treatment group before and after treatment

| Brain Region | R/L | BA | MNI (Peak point) | | | T value | Voxel |
| --- | --- | --- | --- | --- | --- | --- | --- |
|  |  |  | X | Y | Z |  |  |
| precuneus | R | 7 | 3 | -67 | 50 | 7.66 | 15 |
| middle frontal gyrus | R | / | 27 | -7 | 62 | 4.95 | 15 |
| superior temporal gyrus | R | 38 | 30 | 17 | -31 | -8.02 | 17 |

Note: R, right. L, left. MNI, Montreal Neurological Institute. p<0.05, FDR corrected.
