# Supplementary material for: Modulation of Brain Activity and Functional Connectivity by Acupuncture Combined With Donepezil on Mild-to-Moderate Alzheimer's Disease: A Neuroimaging Pilot Study
Source: Front Neurol. 2022 Jul 11;13:912923. doi: 10.3389/fneur.2022.912923 (PMC9309357; doi:10.3389/fneur.2022.912923)
Supplement: Supplementary file 3 [file Table_3.DOCX]

SUPPLE Table 3 Regions showing significant fALFF value changes within the control group before and after treatment

| Brain Region | R/L | BA | MNI (Peak point) | | | T value | Voxel |
| --- | --- | --- | --- | --- | --- | --- | --- |
|  |  |  | X | Y | Z |  |  |
| lingual gyrus | R | / | 9 | -82 | -7 | 8.5 | 10 |
| middle frontal gyrus | R | 9 | 57 | 23 | 35 | -3.76 | 15 |

Note: R, right. L, left. MNI, Montreal Neurological Institute. p<0.05, FDR corrected.
