# Supplementary figures and images for: Modulation of Brain Activity and Functional Connectivity by Acupuncture Combined With Donepezil on Mild-to-Moderate Alzheimer's Disease: A Neuroimaging Pilot Study
Source: Front Neurol. 2022 Jul 11;13:912923. doi: 10.3389/fneur.2022.912923 (PMC9309357; doi:10.3389/fneur.2022.912923)

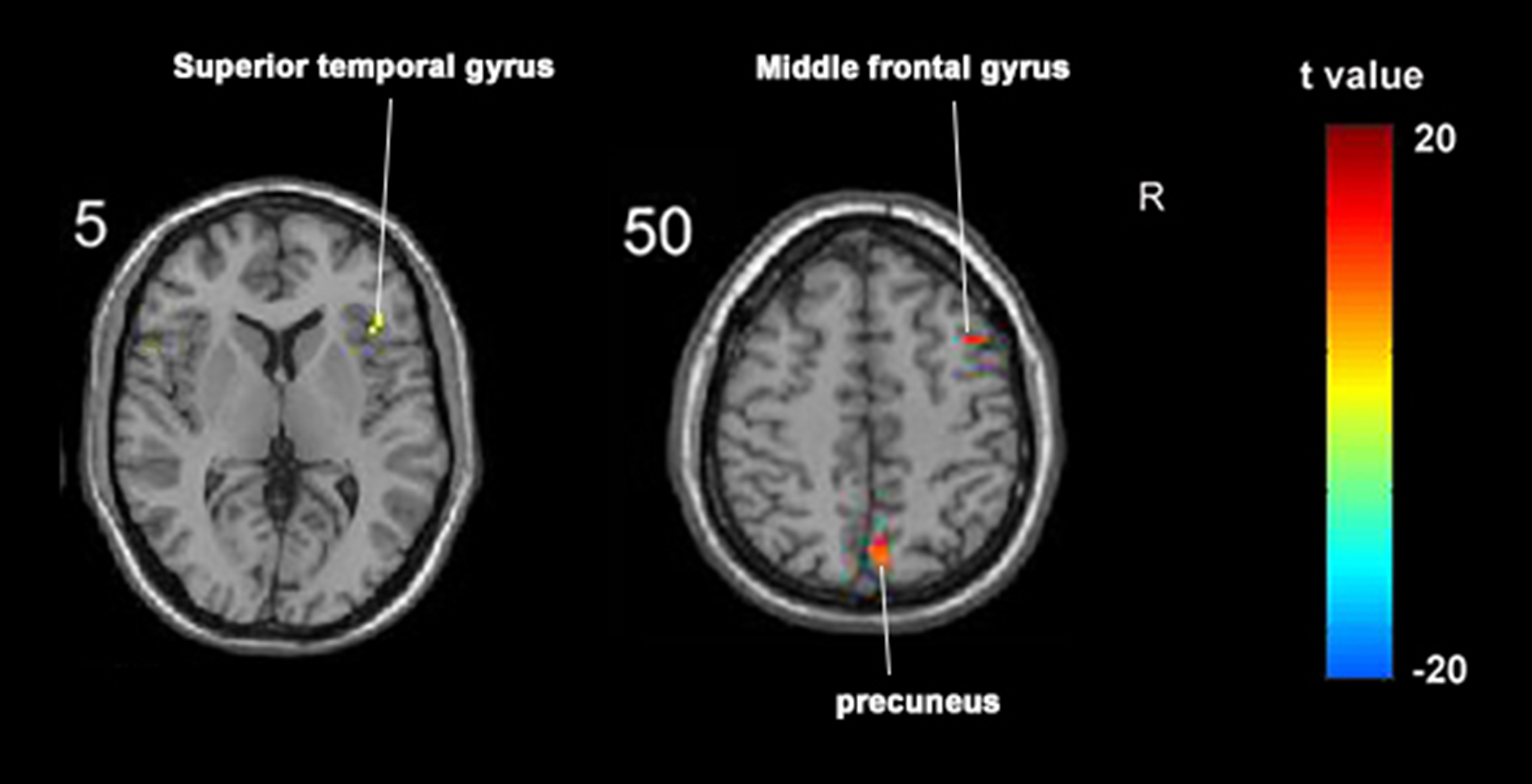

Supplement: Supplementary Figure S1 — Regions showing significant fALFF value changes within the treatment group before and after treatment. The results were corrected with p < 0.05, FDR-corrected. [file Image_1.JPEG]

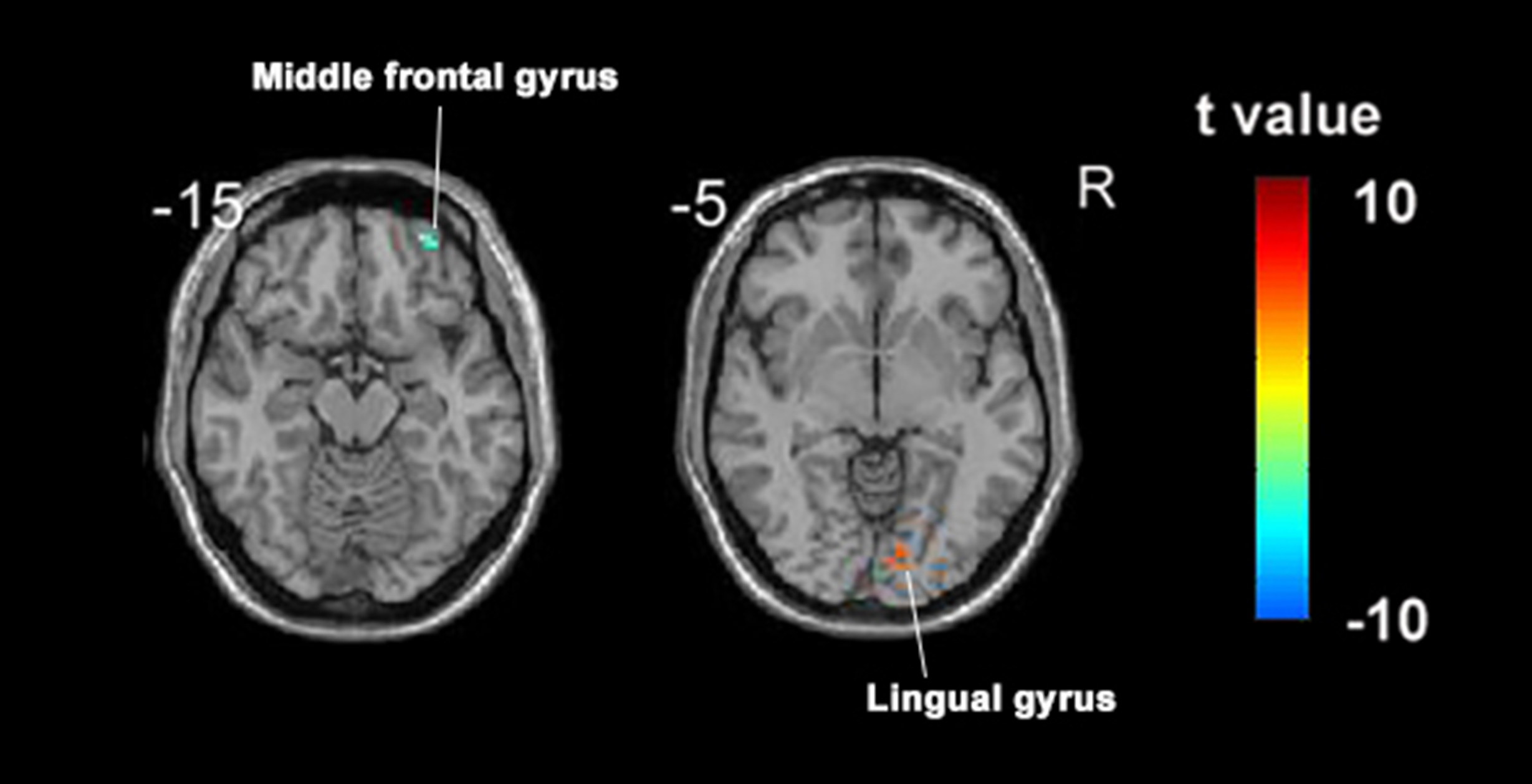

Supplement: Supplementary Figure S2 — Regions showing significant fALFF value changes within the control group before and after treatment. The results were corrected with p < 0.05, FDR-corrected. [file Image_2.JPEG]
